# Self-Supervised Open-Set Speaker Recognition with Laguerre–Voronoi Descriptors

**DOI:** 10.3390/s24061996

**Published:** 2024-03-21

**Authors:** Abu Quwsar Ohi, Marina L. Gavrilova

**Affiliations:** Department of Computer Science, University of Calgary, Calgary, AB T2N1N4, Canada

**Keywords:** representation learning, self-supervised learning, deep neural network, Laguerre–Voronoi diagram, open-set speaker recognition, behavioral biometric, smart sensors

## Abstract

Speaker recognition is a challenging problem in behavioral biometrics that has been rigorously investigated over the last decade. Although numerous supervised closed-set systems inherit the power of deep neural networks, limited studies have been made on open-set speaker recognition. This paper proposes a self-supervised open-set speaker recognition that leverages the geometric properties of speaker distribution for accurate and robust speaker verification. The proposed framework consists of a deep neural network incorporating a wider viewpoint of temporal speech features and Laguerre–Voronoi diagram-based speech feature extraction. The deep neural network is trained with a specialized clustering criterion that only requires positive pairs during training. The experiments validated that the proposed system outperformed current state-of-the-art methods in open-set speaker recognition and cluster representation.

## 1. Introduction

Speech is the most common form of human communication. Speaker recognition is related to human biometrics that identifies humans based on the properties of their voice. Speaker recognition is a key technology in the modern era due to its seamless implementation in voice automation, voice authentication, smart home devices, etc. Microphones integrated with speaker recognition technology serve as intelligent sensors within the Internet of Things ecosystem, enabling them to respond selectively to commands provided by authorized users. Self-supervised learning (SSL) is the process of learning representation from unlabeled data [1,2]. For speaker recognition, SSL offers numerous advantages over supervised learning. SSL can nullify the cost of speech data labeling. The availability of unlabeled data offers instantaneous development and enhancement of learning capabilities. Moreover, SSL addresses privacy concerns that may arise when acquiring labeled speech data in certain situations. Due to its significance and scalability, SSL is frequently used for training speaker-recognition systems.

Open-set speaker recognition is a domain that distinguishes speakers based on speech, regardless of whether they are known/registered or not [3]. In the speaker-recognition domain, the performance of open-set speaker-recognition methods is measured based on the accuracy of user verification [4]. A clusterable open-set speaker-recognition system can not only determine the number of speakers within a given set, but also extract a rich array of additional attributes, such as gender, age, voice characteristics, etc. [5]. Clusterable open-set speaker-recognition systems can further be used for speaker diarization and speaker change detection. In short, clusterable open-set speaker recognition can provide a wealth of information over binary verification, and it can be leveraged in a wide range of applications, including speech personalization, speech profiling, and speech-based interfaces.

Deep neural networks (DNNs) have been extensively investigated for speaker recognition [6], along with the loss functions they are trained with. Yet, DNNs have not been investigated for open-set speaker recognition, and most studies solely focus on altering the loss functions [7,8,9]. As a result, DNNs in the current literature do not utilize speech features to learn speech representation. Speech features can be learned by extracting speech descriptors, which would help identify speech properties. Speech descriptors are characteristic elements of speech such as speed, pitch, tone, intensity, and other highly discriminating features of speech. Therefore, this work proposes an optimal DNN architecture that is specialized for the unsupervised speaker-representation task. Furthermore, instead of altering the loss functions, this work focuses on the training criterion of the DNN to efficiently separate speakers based on speech properties.

This paper proposes a novel DNN-based open-set speaker-recognition system that generates clusterable speaker embeddings. Moreover, the training strategy of the speaker-recognition system does not require labeled data. During the training, the DNN learns the similarity between speech samples with a hand-crafted similarity criterion function. The DNN architecture consists of a mixture of high receptive spatial and channelwise attention blocks. In addition, a unique Laguerre–Voronoi diagram-based speaker-descriptor layer is introduced to improve the clustering ability of the system. The paper contributions are as follows:

- A novel Laguerre–Voronoi descriptor deep neural network (LVDNet) architecture, realizing a self-supervised learning approach, is proposed. The architecture allows operating efficiently with short speech segments.
- A novel Laguerre–Voronoi-based vector of locally aggregated descriptors, namely Laguerre–Voronoi descriptors (LVD), is introduced to extract essential speech features while filtering out noise.
- A unique clustering criterion is developed to help the DNN generate clusterable speaker representation.

To validate the effectiveness of the proposed system, it was extensively compared with some of the recent works in the domain. The VoxCeleb1 [10] and LibriSpeech [11] benchmark datasets were used to validate the method. Apart from open-set performance, verification performance, memory consumption, and the complexity of the DNNs were also examined. Through rigorous investigation, it was validated that the proposed work gives state-of-the-art performance for generating clusterable speaker embeddings.

The rest of the paper is structured as follows: Section 2 presents a review of the works performed in the speaker recognition domain. Section 3 describes the proposed strategy. Section 4 provides an overview of the dataset, metrics, and implementation constraints, followed by experimental results. Finally, Section 5 concludes the paper.

## 2. Related Works

Speaker recognition is one of the cores of biometric recognition methods that has numerous applications. The first speaker-recognition systems were built using statistical machine learning models [12]. The process included training a model for extracting speech embeddings, referred to as speech vectors, using machine learning models. In the second stage, these speech vectors were used to train a classification module. Due to the scalability and robustness, later, aDNN was used to extract the speech vectors [13].

DNNs have demonstrated exceptional performance due to specialized architectures and augmentation strategies [14]. With continuous enhancements, the end-to-end training strategy became robust and incorporated feature extractors [15]. Specialized neural networks were developed to extract speech features from raw waveforms [6]. Further research exploited pooling strategies for the recognition process. The vector of locally aggregated descriptors (VLAD) performed well in supervised speaker recognition tasks as it can extract prominent speech descriptors [16]. Further modification led to GhostVLAD [17], which introduced additional descriptors that filter out the noisy and erroneous features. As speech exhibits a frequency over time, attempts have been made to extract the crucial speech features throughout the temporal sequences. Self-attentive pooling (SAP) [18] applies attention to the speech features extracted over time. Attentive statistics pooling [19] (ASP) expands the idea of attention further by calculating the mean and standard deviation of the attended speech features. Although these pooling strategies perform better than usual poolings, they fail to memorize feature descriptors, which are the key characteristics of the data.

As training a DNN requires a large quantity of labeled data, speaker recognition generative adversarial network-based architectures were also investigated to reduce the need for a large labeled dataset [20]. The success of SSL in language processing and computer vision tasks has also enhanced the speaker-recognition domain. In computer vision, SSL is implemented using Siamese twin networks [21], which involves two networks sharing identical weights. However, speech is continuous, and a conversation usually contains multiple speakers. Therefore, in self-supervised speaker-recognition systems, the common approach is to separate continuous speech into segments (based on voice activity, speaker transition, or random slicing) and attributing each segment to a particular speaker [22]. In this strategy, the DNN remains unaware of the number of classes since each segment is assigned a pseudo-label. It is worth noting that most SSL-based speaker-recognition methods use a 1.8 s speech length [4]. Although using longer speech lengths may increase the performance of the models, frequently, it is unattainable to fetch longer speech segments as this also increases the possibility of having multiple speakers [23]. Therefore, developing robust open-set speech-recognition approaches that can be used with shorter speech segments is essential.

The concept of open-set recognition involves training a model to learn the data variations (similarity and dissimilarity), which also aligns with the training process of SSL. Therefore, most of the current speaker-recognition approaches target open-set recognition. Chung et al. [4] explored diverse DNN models for open-set speaker recognition in conjunction with multiple loss functions and confirmed the efficiency of DNNs in verification tasks. However, the training involved labeled data, and the method requires large batch sizes as it gives a broader spectrum of variation during training. Mun et al. proposed Contrastive Equilibrium Learning (CEL) [24], which works in open-set speaker recognition trained in a self-supervised manner. However, as the system focuses on speaker similarity learning, it does not perform equally on speaker identification tasks.

SimSiam has an SSL architecture that is one of the minimalist one, which can learn similarity. Sang et al. [8] proposed a regularized version of SimSiam for open-set speaker recognition. Apart from the default loss proposed in SimSiam, the method inherits angular prototypical loss. The regularization loss is the negative of the unit length to the end of the encoder to emphasize similarity learning. As the system solely focuses on similarity learning, the network loses its balance upon learning dissimilarities. The approach proposed in [7] adapts SimSiam for producing clusterable speech embeddings. Along with SimSiam’s loss, the method combines prototypical loss and cosine distance loss. Although the method produces clusterable embeddings, it relies on a straightforward DNN architecture, which limits its performance.

Self-distillation with no labels (DINO) [25] is a popular SSL method that inherits the dynamism of vision transformers [26]. DINO produces remarkable performance in unsupervised object segmentation. Due to its popularity, it also has been implemented in open-set speaker recognition. Han et al. [27] introduced a DINO-based framework that required two-stage training. The first stage includes training the DINO framework. In the second stage, the DNN is trained with cluster relationships produced by its embedding in the first stage. Two-stage training is often a hurdle as the second stage relies on the outcome of the first stage. The work of [9] further introduces a DINO-based approach that requires single-stage training. However, instead of vision transformers, the method uses a time-delay network [28], which limits the effectiveness of DINO.

As seen from the above discussion, the prior research works lacked exploiting an independent training strategy, relied on labeled data, and could not handle short speech segments. As a result, prior research studies were confined by proposing regularization terms with training baselines. Moreover, to the best of our knowledge, there is no previous work that investigates DNN strategies tailored to speaker representation learning. The aforementioned research gaps motivated us to propose a stand-alone concise training strategy for open-set speaker recognition. Moreover, this work also bridges the gap of studies required for the DNN architecture and descriptor networks.

## 3. Methodology

This work introduces a self-supervised open-set speaker-recognition system that transforms speech into clusterable embeddings. The system employs a novel Laguerre–Voronoi descriptor deep neural network (LVDNet) architecture featuring spatial and channelwise attention mechanisms. Additionally, LVDNet leverages dilated convolution to achieve broad coverage of temporal speech features that help the system locate a wider dependency on temporal speech features. The LVDNet-extracted speech features undergo additional processing through an exceptional Laguerre–Voronoi descriptor (LVD) layer. The LVD layer identifies the most frequently occurring speaker-dependent features and generates speaker embeddings accordingly. Additionally, the LVD layer effectively filters out common features that could degrade the quality of the speaker embeddings. The extracted speaker embeddings are further processed by an efficient cluster criterion function that allows the speaker embeddings of the same speaker to be slightly distant based on speech variability. The adaptability of this clustering criterion empowers the model to acquire enhanced speech representations, leading to improved recognition performance.

**Figure 1:**
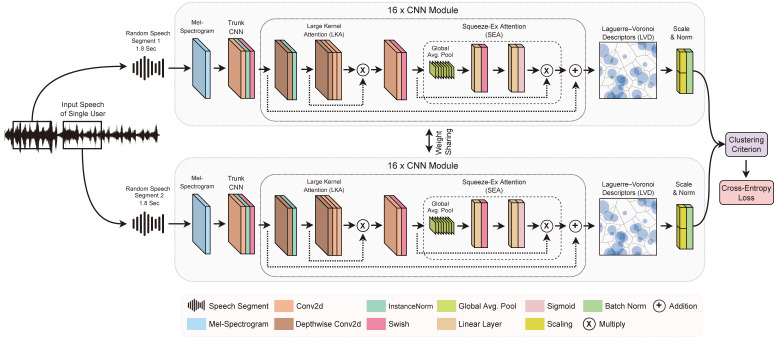
Overall training architecture of the open-set self-supervised LVDNet for speaker recognition.

The proposed framework is self-supervised and capable of open-set recognition. As a result, it can be trained using an unlabeled speech dataset and then applied to recognize speakers across various datasets. Similar to the prior works [8,9,24], it is assumed that the model is trained with a dataset, where each piece of audio contains the speech of only one person. For each audio piece, two random segments from the audio speech is selected to train the LVDNet model in every epoch. The LVDNet model is fed with the pair of audio pieces while training, where the task is to produce similar (not effectively exact) speech embedding for the same speakers. Figure 1 explains the training strategy along with the proposed network architecture. The following subsections sequentially introduce the LVDNet architecture, the Laguerre–Voronoi descriptor, and the cluster criterion.

### 3.1. Laguerre–Voronoi Descriptor Deep Neural Network (LVDNet) Architecture

The LVDNet consists of a ResNet-like skeleton with several modifications. The backbone uses swish [29] as the default activation function as it provides a smoother gradient. The backbone also uses instance normalization [30]. Instance normalization eliminates instance-specific information, such as noise, from speech, leading to a streamlined generation process. Batch normalization [31] is used at the end of the output, which speeds up the convergence. The normalized log-Mel spectrogram is first passed through a standard convolution with kernel size 7 and 16 channels, followed by a group norm and swish activation. Subsequently, the network involves {3, 4, 6, 3} CNN modules, each utilizing a channel size of {16, 32, 64, 128} for all convolutions in the respective module. The number of CNN modules and channel sizes were interpolated from popular speaker-recognition architectures [4,32].

Each CNN module consists of a depthwise convolution, normalization, large-kernel attention (LKA) [33], convolution, activation, and a squeeze–excitation attention (SEA) [34] layer, respectively. Figure 1 depicts the architecture for each convolutional block. In the following two sections, the LKA and SEA layers are derived.

#### 3.1.1. Large-Kernel Attention (LKA)

The Mel spectrogram explains the human-audible frequencies that vary with time. As an audible sound contains a mixture of multiple frequencies, a large receptive field can extract the dependency within a wide range of frequency bins with respect to the time domain. In the current literature, time-delay neural networks (TDNNs) [35] extract long-term temporal dependencies. However, due to the sub-sampling of the TDNNs, they lose temporal information.

**Figure 2:**
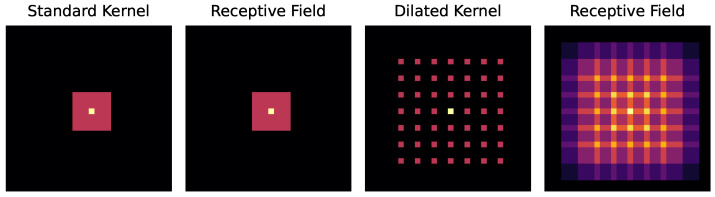
The standard kernel and its receptive field, followed by the dilated kernel and its receptive field.

The LKA module consists of a depthwise convolution, followed by a convolutional layer with a kernel size of 7 with a dilation of 3. The dilated convolution is followed by a pointwise convolution layer. Due to the dilation and higher kernel size, the LKA has a larger receptive field than the standard convolution (illustrated in Figure 2). Therefore, the LKA module can cover long-range spatial (temporal and frequency) dependency while keeping spatial and channel adaptability. Hence, it can extract a broad dependency of frequency based on time. The LKA extracts an attention map from the feature map with the set of depthwise and pointwise convolutions. Equation (1) formally defines the process [33].

(1) $$M_{attention}=Conv_{Pointwise}\left(DialatedConv_{DepthWise}\left(Conv_{DepthWise}\left(F\right)\right)\right)$$

(2) $$Output=M_{attention}⊗F$$

The receptive field attention map is further used to weight the input feature map, which is the final output of the LKA function. Equation (2) defines the attention weighting procedure, where ⊗ is an elementwise multiplication operation.

#### 3.1.2. Squeeze–Excitation Attention (SEA)

The first linear layer in SEA downsamples the channels to half, followed by another linear layer that upsamples the channels to the previous value. The linear layer concentrates on the inter-channel features and recalibrates the channelwise feature representations based on interdependencies.

### 3.2. Laguerre–Voronoi Descriptors (LVDs)

Pooling is used for down-sampling the features of a DNN. In contrast, descriptors capture regions in the embedding space and help extract discriminative features from the input. The distance from a descriptor computes the intensity of the presence of the particular feature. As speech has various properties (depth, hardness, softness, and so on), descriptors can extract the intensity of the properties. Therefore, the combination of the intensity of various speech features or descriptors can generate a speech representation in a high-dimensional embedding space. As these features are speaker-dependent, the representation should be clusterable. The intuition behind introducing the LVD as a weighted parameter is its remarkable ability to region speech properties in the vector space. Moreover, the LVD can dynamically adjust the number of features by down-scaling the number of regions in the vector space [36].

A Voronoi diagram consists of *n* centroids and partitions a plane onto *n* regions using convex polygons. The regions are built based on the shortest distance between the generating centroids to any other point in that polygon. Let $c_{i}$ be the centroids, then the Voronoi polygon of centroid $c_{i}\in R^{d}$ can be defined as [36]:

(3) $$V\left(c_{i}\right)=\cap_{j}\left\{p\in R^{d}|D\left(c_{i},p\right)\leq D\left(c_{j},p\right)\right\}$$

Here, D(·,·) is the distance function, which calculates the distance between the centroid $c_{i}$ and any other point *p*.

In Laguerre geometry, the centroids are considered as a circle $C_{i}=\left\{c_{i},r_{i}\right\}$ with $r_{i}\in R$ radius and $c_{i}\in R^{d}$ as the centroids. The distance between a circle $C_{i}$ and a point *p* is defined as [36]:

(4) $$P\left(C_{i},p\right)=\left|\right|c_{i}-p{\left|\right|}_{2}^{2}-r_{i}^{2}$$

Based on Equation (4), the Laguerre–Voronoi polygon for circle $C_{i}$ is defined as:

(5) $$V_{l}\left(C_{i}\right)=\cap_{j}\left\{p\in R^{d}|P\left(C_{i},p\right)\leq P\left(C_{j},p\right)\right\}$$

**Figure 3:**
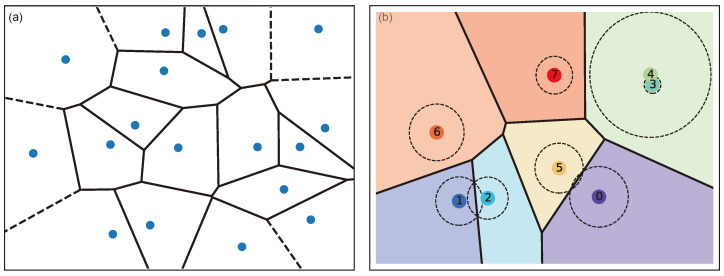
(**a**) Voronoi diagram and (**b**) Laguerre–Voronoi diagram in 2D.

Here, P(·,·) is the power distance function in Laguerre geometry, defined in Equation (4). Figure 3 illustrates the Voronoi diagram and the Laguerre–Voronoi diagram.

The LVD follows the properties of Laguerre geometry. Therefore, the intensity of the descriptor is determined by the power equation derived in (4). Moreover, the descriptors built their own Laguerre polygon with respect to their radius and centroids. The radius and centroids of the descriptors are trainable parameters, which are learned through backpropagation. The radius value $r_{i}$ serves as a weighting factor, which exerts control over the area of the enclosed polygon, $V_{l}\left(C_{i}\right)$. A decrease in the radius of a circle can result in a reduction of the polygon’s area, and in cases of exceedingly low radii, the polygon may cease to exist. Figure 3b illustrates an instance where centroid 3 exhibits no area coverage because of its smaller radius. This property of the LVD helps to set only an upper limit of the descriptors, which can dynamically be reduced if necessary. Apart from descriptors, we also added some additional centroids to the LVD to filter some most common distractors. Distractors are some of the common impurities in sound that can cause performance degradation in speaker representation learning (noise, room impulse response, etc.).

**Figure 4:**
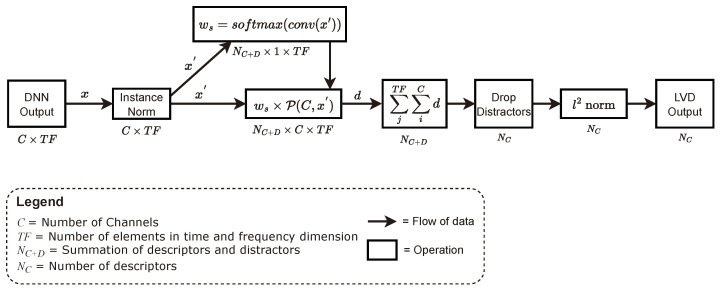
Flow chart of the proposed Laguerre–Voronoi descriptor (LVD) layer. The output dimension of each operation is given below the operations.

Figure 4 depicts the workflow of the LVD. The output of LVDNet, a three-dimensional feature map $F_{C\times T\times F}$, undergoes the merging of the temporal and frequency dimensions, $F_{C\times TF}$. Instance normalization is applied to the merged feature, $F_{C\times TF}$. Further weight $w_{s}$ is calculated from the normalized input by performing convolution followed by a softmax function. The weight is used to scale the power distance $P(C,x^{\prime })$ between the feature maps and each of the centroids. Centroids dynamically converge to the locations where various important speech properties are congregated in the embedding vector space. The power distance function calculates the distance of the features from the learnable centroids. The power distance from the feature maps to the centroids indicates how well a feature holds a certain property of speech. There are a total of C+D centroids, where *C* is the number of speech descriptors and *D* is the number of distractor descriptors. While the speech descriptors try to find the quality or intensity of certain speech characteristics from the feature map, distractors identify the impure features in the feature vector space. The dimension of the power distance is reduced by summing over the channel axis and the temporal frequency axis. As a result, the output $F_{N_{C+D}}$ indicates the intensity of each speech descriptor and distractor. As the distractor centroids only find the intensity of common irrelevant features, they are dropped from the final features. Finally, the centroid descriptor is normalized.

### 3.3. Scaling

After the pooling, the embedding is passed through a scaling layer, which acts as the final output from the model. The scaling layer consists of two linear layers, which can be derived as follows:

(6) $$S\left(x\right)=\left(w_{1}x+b_{1}\right)+e^{0.1\times (w_{2}x+b_{2})}$$

Here, $x_{1}$, $x_{2}$, $b_{1}$, and $b_{2}$ are the weights and biases for the first and second linear layers, respectively. Due to the exponent value, the scaling function can output a wide range of values with a subtle weight shift in the network. It helps the network to cover a large embedding sphere.

### 3.4. Cluster Criterion

A typical training strategy for speaker recognition involves reducing the cosine distance between two speech segments for similar speakers. However, for speech representation learning, it is not always true that two speech segments should have the exact representation, because speech representation may contain slight changes due to the speech pattern, emotion, and other factors. Therefore, training a DNN to output exact speech embeddings based on the speaker is not intuitive. This issue is addressed by adding a marginal function to give a threshold of similarity between the cosine distance between two speech embeddings. The margin function produces a similarity score [0,1], where a higher score means higher similarity. The margin function can be derived as

(7) $$M\left(d\right)=\frac{w}{1+e^{-d\times c_{1}+c_{2}}}$$

Here, *d* is the cosine distance between the embeddings of two speech segments. *w* is a trainable variable, which scales the margin function based on the loss function. The value of *d* is a real number in the range [−1,1], and a higher value maps to a higher similarity between two embeddings. Equation (7) is a modification of the Sigmoid activation function $\frac{1}{1+e^{-x}}$, which is down-scaled and shifted to meet the requirement of the clustering criterion. The equation consists of two constant variables, $c_{1}$ and $c_{2}$. The value of $c_{1}$ is proportional to the shrinkage of the Sigmoid function. In addition, by increasing $c_{2}$, the Sigmoid activation function can be shifted towards positive with respect to the input, *d*. In Equation (7), the value is set to $c_{1}=8.19$ and $c_{2}=1.95$. In Equation (7), $c_{1}$, $c_{2}$, and $c_{3}$ are constant variables, and the value is set to $c_{1}=1.3$, $c_{2}=1.5$, and $c_{3}=-6.3$. Equation (7) is a modification of the Sigmoid function $\frac{1}{1+e^{-x}}$, which is down-scaled and shifted to meet the requirement of the clustering criterion.

**Figure 5:**
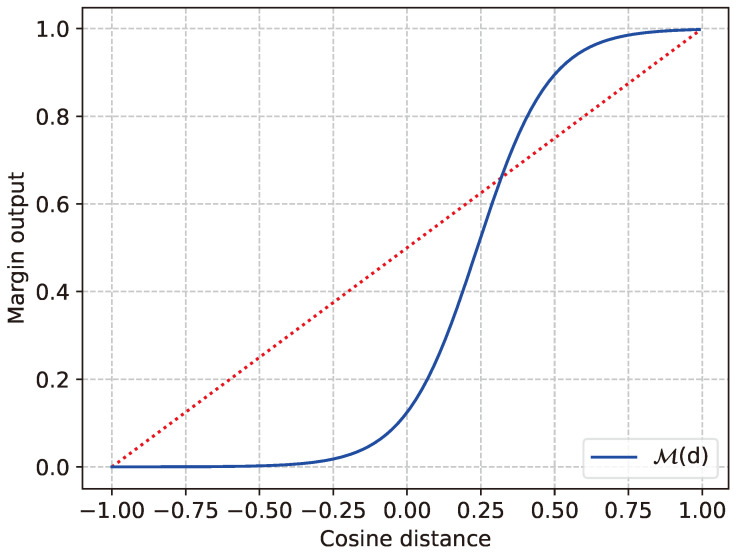
Output of the margin function M(·) with respect to cosine distance (when *w* = 1 in Equation (7)).

Figure 5 illustrates the function’s behavior, when w=1. The margin function generates a value closer to 1 as the output when the cosine similarity of two data points is above 0.75, which adds flexibility to the model to understand diverse speech patterns for an individual. Moreover, the margin function generates an output closer to 0 when the cosine distance is less than −0.25. This limit gives a tighter boundary, which restricts the model to avoid higher cosine distances for similar speech embeddings.

This work introduces a novel LVD layer, which can be trained using backpropagation. To relay the descriptive feature space to the LVD layer, a unique DNN architecture, LVDNet, is introduced. LVDNet can cover a wide area of temporal speech features, which facilitate the network to extract meaningful speech embeddings. Further, an effective clustering criterion is introduced to train the LVDNet without requiring dissimilar data pairs, which refer to the knowledge of a pair of data belonging to different classes. The proposed architecture is validated in the next section.

## 4. Experimental Results

### 4.1. Datasets and Experimental Setup

The proposed architecture was trained on the VoxCeleb1 [10] benchmark dataset containing 1251 speakers in total. The dataset has a training/testing split of 1211 and 40 speakers, respectively. Although VoxCeleb1 includes labels for each of the audio files, they were not considered during training. Only the development training set was used to train the model. The LibriSpeech [11] dataset has been used to perform the open-set recognition on unknown speakers. The test-clean subset of the LibriSpeech (40 speakers) was used to evaluate open-set recognition in a controlled environment. Moreover, the test-other subset of LibriSpeech (33 speakers) was used to evaluate recognition in a noisy environment. For verification on LibriSpeech, ten random samples were chosen for each of the data, with five similar and five dissimilar pairs.

The model was trained using a batch size of 128 for 300 epochs. All audio segments were 1.8 s in length, and they were converted into a log-Mel spectrogram using 40 Mel filter banks with a 25 ms frame length and 10 ms shift. Although the VoxCeleb dataset has real-world noises, more noise was randomly added to the audio for better generalization. The Musan [37] noise dataset was used for this purpose. The overall training was performed using the Adam optimizer with a learning rate of 0.001, with automatic weight decay [38]. All of the methods were trained and tested under the same dataset constraints, without changing the hyperparameters. The DL architectures were trained on an NVIDIA GeForce RTX 3090 with 64 gigabytes of random access memory. The models were re-implemented using pytorch in the python language.

For comparison, CEL [24], SimSiamReg [8], C-SimSiam [7], and DINO-Reg [9] were implemented and trained using the optimal parameters that were suggested by the investigators who proposed the models. However, for DINO-Reg, 3-second and 2-second speech segments were used as the long and short segments, respectively.

The experiments were conducted to demonstrate the clustering performance, which indicates better representation learning, along with verification performance. The Adjusted Rand Index (ARI) is a metric that explains how well particular data are clustered. ARI is a measure of the similarity between two clustering results. It compares all pairs of samples between the clustering assignments and labels and adjusts for random clustering. A higher score indicates the purity of clustering. To calculate the ARI score on unlabeled data, the speaker embeddings are labeled depending on their cluster regions by utilizing k-means clustering. Further, the Equal Error Rate (EER) metric was used to justify the verification performance of the systems. An equal error rate is the threshold of a system where the false acceptance rate and false rejection rates are equal. The output range of the EER is from 0 to 1. A lower score indicates better performance. Memory consumption and inference time are prominent questions for most DNNs. Therefore, the number of parameters of the DNNs is reported to highlight memory consumption. The number of multiplication and accumulation (MAC) operations for the DNNs is also reported to indicate the computational intensity of the DNNs.

### 4.2. Ablation Study

In the ablation study, the effectiveness of the various proposed components was evaluated. LVDNet consists of four major components: (a) large-kernel attention (LKA) + squeeze–excitation attention (SEA): the spatial and channel attention module; (b) the margin constraint during training; (c) the scaling layer at the final output of the model; and (d) the LVD. Table 1 and Table 2 demonstrate the clustering performance and verification performance, respectively. LVDNet achieved 94.98% clustering performance on VoxCeleb1 and a 12.87% error rate for verification. Removing the LKA and SEA layers dropped the clustering performance by 10% and increased the verification error by 6.29%. The LKA and SEA layers enable the DNN to have a wider receptive field, which helps extract more diverse temporal speech features. Removing the LKA and SEA limits the receptive field over temporal speech features. The margin constraint gives more stability in the representation of the speech embeddings, as it is not strict about producing exact embeddings for two different speech frames. Therefore, removing the margin function increased the verification error by 5.27%. The scaling layer enables the model to generate a wide range of embedding points, facilitating the creation of distinct cluster regions. The LVD generates the intensity of speech features while filtering out recurring distractive features. Replacing the LVD with basic average pooling increased the verification error by 4.18% and reduced the clustering performance by 4.9%.

**Figure 6:**
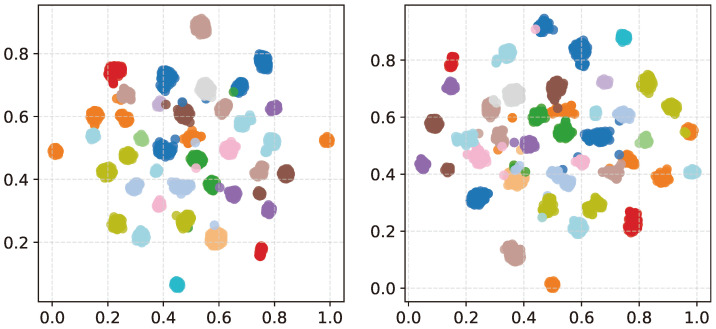
Speech embeddings with (**left**) and without margin function (**right**).

Figure 6 illustrates a 2D scatter plot of speech embeddings produced from the DNN when trained with and without the margin function. The margin function does not tightly bind similar speech embeddings. Relaxing the similarity constraint helps the model to perform the embedding while training. The motion of embeddings allows the model to condense the most similar embeddings over time. Therefore, embeddings of similar speech properties end up in a global optimum cluster after the training. Without the margin function, the embeddings cannot converge toward the optimal cluster point. As a result, cluster regions have irrelevant/noisy embeddings when trained without a margin function.

### 4.3. Comparison of Feature-Aggregation Strategies

The Laguerre–Voronoi descriptor is one of the major contributions of LVDNet. Therefore, the LVD was compared with the traditional feature aggregation strategies used in the speaker-recognition domain, with the results presented in Table 3. Average pooling aggregates features by the statistical mean. Therefore, it cannot discriminate between time and frequency. As a result, it reduces the verification and clustering performance by approximately 4% compared to the proposed LVD. SAP [18] incorporates attention to weight over the temporal axis of the speech feature, highlighting the most crucial speech frequencies over time. However, as it does not identify major speech features, it reduced the clustering and verification performance by 2% compared to the proposed LVD model. ASP [19] operates similarly to SAP in the case of temporal attention. Yet, ASP adds an attentive deviation of features over time. However, its inclusion did not improve the clustering and verification performance. GhostVLAD [17] learns speech descriptors and returns the residual between learnable descriptor centroids and the embedding. As residuals are not unique in magnitude and the number of centroids/descriptors is not dynamically adjusted, GhostVLAD cannot generate proper speech embedding. Compared to other feature-aggregation strategies, the LVD calculates the speech descriptors irrelevant with respect to time and provides the intensity of the features. Moreover, the LVD can dynamically decrease the centroid radius when necessary. Hence, it can output embeddings based on speech properties, which are also clusterable.

### 4.4. Comparison of DNN Models

Table 4 represents a comparison of the proposed LVDNet with different DNN architectures. DNN architectures from the recent speaker-recognition frameworks ThinResNet [32], FastResNet [4], the time-delay neural network (ECAPA-TDNN) [28], and C-SimSiam [7] were used in the experimentation. ThinResNet and FastResNet are the lighter implementations of the ResNet architecture and are nearly similar. Therefore, their performance in clustering and verification was identical. However, as ResNet was solely designed for computer vision tasks, its successor models fell behind by roughly 3% in verification and clustering performance. ECAPA-TDNN was designed for speech-processing tasks and has a wider viewpoint over speech features due to higher dilation. However, as ECAPA-TDNN reduces features with each layer, the temporal information is also lost within. Therefore, ECAPA-TDNN loses its performance by around 4% on the LibriSpeech dataset. C-SimSiam inherits FastResNet while reducing the number of parameters and having different activation functions. The changes provide better generalization. Yet, the DNN fails to correlate temporal features with the frequency distribution. Hence, the performance of the DNN degrades by 2% compared to the proposed DNN.

**Figure 7:**
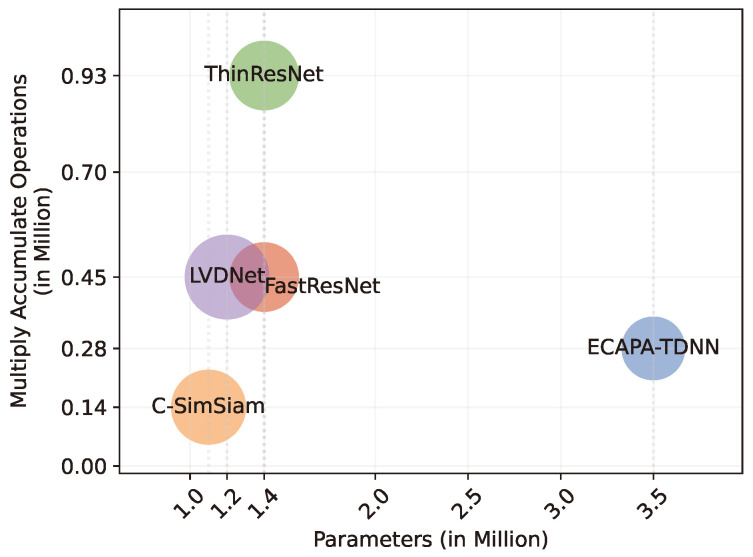
Comparison of DNN models with respect to parameters, MAC operations, and performance.

Figure 7 exhibits the number of parameters (in the x-axis) and the number of MAC operations (in the y-axis). The MAC operations for the DNN are reported for 1.8 s of audio data. Each circle represents the different DNNs presented in Table 4. The region covered by the circles indicates the performance of the model. C-SimSiam consumes the least memory, having only 1.1 million parameters and 0.14 million MAC operations. Both ThinResNet and FastResNet have 1.4 million parameters. However, FastResNet performs 0.45 million MAC operations, while ThinResNet computes 0.93 million MAC operations. ECPA-TDNN is the most memory-consuming DNN, having 3.5 million parameters and requiring 0.28 million MAC operations. Compared to the existing DNNs the proposed DNN architecture requires 1.2 million parameters. Moreover, it requires 0.45 million MAC operations.

### 4.5. Comparison with Other Methods

Table 5 contains the comparison of LVDNet with the existing self-supervised approaches. In the comparison, DINO-Reg used a maximum of 3 s speech intervals during training. However, it gave a notable difference in the EER and ARI scores while showing degraded performance. Although DINO is an excellent method, in the case of speaker recognition, it fell behind due to the requirement of heavily tuning its hyperparameters. CEL and SimSiamReg do not have heavy hyperparameter tuning. However, these methods fell behind in clustering performance (ARI) while giving considerable verification performance (EER). This difference in verification and clustering performance verifies that, although a model can perform better in verification, it might not perform better in identification. Further, C-SimSiam emphasizes generating clusterable embeddings. Therefore, it generated a balanced performance for verification and clustering/identification. Compared to other methods, the proposed framework not only performed better, but also gave balanced results for the EER and ARI.

**Figure 8:**
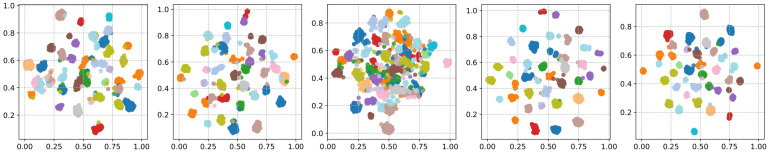
Speech embeddings generated by CEL, SimSiamReg, DINO, C-SimSiam, and LVDNet.

Figure 8 further illustrates the two-dimensional cluster embeddings of the LibriSpeech-clean subset. The dimensions of the cluster embeddings were mapped to a two-dimensional space using the T-SNE. The embeddings of DINO are not clusterable. Furthermore, CEL and SimSiamReg provided more clusterable embeddings containing cluster overlaps and noises. Compared to CEL and SimSiamReg, C-SimSiam generated better and cleaner embeddings while having noises in the clusters. Finally, the proposed method contained less noise and tighter cluster regions.

## 5. Conclusions

This paper proposes a novel open-set self-supervised speaker-representation model. The method performs better than its competitors due to the minimal clustering criterion. Moreover, the advantage of the model lies within the Laguerre–Voronoi diagram-based speaker descriptor pooling strategy, along with a deep neural network, which can extract broader temporal speech features. Through extensive comparison with the state-of-the-art works, it was established that the proposed method outperformed the other methods on two benchmark datasets in both speech verification and identification tasks. In the future, the architecture can be implemented for speaker diarization problems.

## Figures and Tables

**Table 1:** Clustering performance of LVDNet by excluding different components.

Model Components				Clustering Performance (ARI ↑)		
**LKA + SEA**	**Margin**	**Scaling**	**LVD**	**VoxCeleb1**	**LibriSpeech-Clean**	**LibriSpeech-Other**
**✗**	**✓**	**✓**	**✓**	84.98	92.33	91.97
**✓**	**✗**	**✓**	**✓**	91.43	84.58	90.47
**✓**	**✓**	**✗**	**✓**	92.67	92.76	94.33
**✓**	**✓**	**✓**	**✗**	90.08	90.32	89.61
**✓**	**✓**	**✓**	**✓**	94.98	94.93	95.43

**Table 2:** Verification performance of LVDNet by excluding different components.

Model Components				Verification Performance (EER ↓)		
**LKA + SEA**	**Margin**	**Scaling**	**LVD**	**VoxCeleb1**	**LibriSpeech-Clean**	**LibriSpeech-Other**
**✗**	**✓**	**✓**	**✓**	19.16	8.27	7.54
**✓**	**✗**	**✓**	**✓**	18.14	8.18	7.79
**✓**	**✓**	**✗**	**✓**	17.42	9.38	9.33
**✓**	**✓**	**✓**	**✗**	17.05	12.08	12.35
**✓**	**✓**	**✓**	**✓**	12.87	7.66	6.26

**Table 3:** Performance benchmark using different feature-aggregation strategies. ↑ indicates higher value is better, ↓ indicates lower value is better.

Feature-Aggregation Strategy	Performance					
	**VoxCeleb1**		**LibriSpeech-Clean**		**LibriSpeech-Other**	
	**EER (↓)**	**ARI (↑)**	**EER (↓)**	**ARI (↑)**	**EER (↓)**	**ARI (↑)**
Avg-Pooling	17.05	90.08	12.08	90.32	12.35	89.61
SAP [18]	14.77	92.07	9.66	92.03	9.59	92.35
ASP [19]	15.66	91.40	10.41	91.43	8.83	92.44
Ghost-VLAD [17]	17.05	90.08	8.04	92.32	8.35	92.61
LVD	12.87	94.98	7.66	94.93	6.26	95.43

**Table 4:** Performance comparison of different DNN models with LVDNet. ↑ indicates higher value is better, ↓ indicates lower value is better.

DNN Models	Performance						Parameters (in Millions)	Multiply Accumulate Operations (in Millions)
	**VoxCeleb1**		**LibriSpeech-Clean**		**LibriSpeech-Other**			
	**EER (↓)**	**ARI (↑)**	**EER (↓)**	**ARI (↑)**	**EER (↓)**	**ARI (↑)**		
ThinResNet [32]	14.88	90.29	10.28	91.28	9.8	92.40	1.4	0.93
FastResNet [4]	14.55	90.37	10.37	91.23	9.83	92.43	1.4	0.45
ECAPA-TDNN [28]	14.86	90.98	11.05	90.71	12.38	90.26	3.5	0.28
C-SimSiam [7]	14.79	91.76	9.86	92.47	8.88	93.55	1.1	0.14
LVDNet	12.87	94.98	7.66	94.93	6.26	95.43	1.2	0.45

**Table 5:** Comparison of different self-supervised open-set speaker-recognition methods. ↑ indicates higher value is better, ↓ indicates lower value is better.

Methods	Speech Seg.	Performance					
		**VoxCeleb**		**LibriSpeech-Clean**		**LibriSpeech-Other**	
		**EER (↓)**	**ARI (↑)**	**EER (↓)**	**ARI (↑)**	**EER (↓)**	**ARI (↑)**
DINO-Reg [9]	3.0	29.61	48.05	26.04	45.79	25.22	55.19
CEL [24]	1.8	17.8	83.85	10.7	84.69	9.92	87.95
SimSiamReg [24]	1.8	19.33	83.92	10.03	82.86	9.98	88.36
C-SimSiam [7]	1.8	15.4	90.26	9.87	90.65	9.09	90.38
LVDNet	1.8	12.87	94.98	7.66	94.93	6.26	95.43

## Data Availability

Data are contained within the article.

## Abbreviations

The following abbreviations are used in this manuscript:

SSL	self-supervised Learning
DNN	deep neural network
LVD	Laguerre–Voronoi descriptors
LVDNet	Laguerre–Voronoi descriptor deep neural network
VLAD	vector of locally aggregated descriptors
SAP	self-attentive pooling
ASP	attentive statistics pooling
CEL	Contrastive Equilibrium Learning
DINO	self-distillation with no Labels
LKA	large-kernel attention
SEA	squeeze0-excitation attention
TDNN	time-delay neural network
